# Voluntary private health insurance, health-related behaviours and health outcomes: evidence from Russia

**DOI:** 10.1007/s10198-020-01252-2

**Published:** 2020-12-23

**Authors:** Andrey Aistov, Ekaterina Aleksandrova, Christopher J. Gerry

**Affiliations:** 1 National Research University Higher School of Economics, Nizhny Novgorod, Russian Federation; 2 Centre for Health Economics, Management, and Policy, National Research University Higher School of Economics, St. Petersburg, Russian Federation; 3 Oxford School of Global and Area Studies, University of Oxford, Oxford, England

**Keywords:** Moral hazard, Supplemental voluntary health insurance, Health behaviours, Health outcomes, RLMS-HSE, Russia, I130, C250

## Abstract

This paper contributes to the discussion around ex-post (increased utilisation of health care) and ex-ante (changes in health behaviours) moral hazard in supplemental private health insurance. Applying a range of methodologies to data from the Russian Longitudinal Monitoring Survey—Higher School of Economics we exploit a selection mechanism in the data to compare the impact of workplace provided and individually purchased supplemental health insurance on the utilisation of health care, on a range of health behaviours and on self-assessed health. We find compelling policy-relevant evidence of ex-post moral hazard that confirms a theoretical prediction and empirical regularity found in other settings. In contrast to other empirical findings though, our data reveals evidence of ex-ante moral hazard demonstrated by clear behavioural differences between those with self-funded supplemental health insurance and those for whom the workplace finances the additional insurance. We find no evidence that either form of insurance is related to improved self-assessed health.

## Introduction

A wealth of empirical evidence confirms the theoretical prediction that health insurance lowers the price of utilising health care at the point of access and, therefore, gives rise to increased health care consumption. In the absence of being able to perfectly observe individuals’ motives, behaviours or actions, the empirical literature has to overcome the challenge that health insurance arrangements also give rise to adverse selection and to various realisations of moral hazard. The RAND [1] and Oregon [2] Health Insurance Experiments, in the United States, have prompted numerous empirical studies which have been able to robustly identify the relationship between health insurance and health care utilisation by drawing on evidence from experimental settings. Nevertheless, these experiments have not resulted in a consensus concerning the, less theoretically clear, link between health insurance, health outcomes and other forms of health behaviour. A related and swiftly evolving body of literature has sought further empirical settings and methodologies to explore the relationships between health insurance, health behaviours and health outcomes in countries other than the United States and with different health care systems.

This paper adds to that literature, seeking to better understand the complex relationships between health insurance and health-related outcomes by examining longitudinal data from the Russian Federation. In the spirit of many European health care systems, Russian health care is financed through a mandatory health insurance (MHI) system, which—in principle—offers comprehensive health care to Russian citizens, free at the point of delivery. In reality, the system is seriously under-financed, the care—which is of variable quality—is far from comprehensive, and access to it is characterised by regional, demographic and socio-economic inequalities related to the individuals’ capacity to make out-of-pocket payments. Against this background, for the minority who could afford it, a private health insurance system emerged in the 1990s to supplement the mandatory system. More recently, as supplemental private insurance has grown in popularity and increasingly been offered through the workplace, policy discussions have focused on whether and to what extent supplemental health insurance can be an active tool for addressing the short-comings and financial constraints inherent to the current system.

We draw on longitudinal data from the Russian Longitudinal Monitoring Survey—Higher School of Economics (RLMS-HSE), which allows us to attenuate the problem of adverse selection by distinguishing between individuals who self-select supplemental health insurance and those that have it provided for them through their place of employment. As we explain later on, it is improbable in Russia, and empirically unsupported, that respondents would choose their employment based on the prospect of their employer providing a supplemental insurance cover. Therefore, using this framework of insurance ‘types’, we can explore the impact that supplemental health insurance has on health care access, good and bad health behaviours and self-assessed health status. We provide, to our knowledge, the first such study focusing on Russia, we distinguish between ex-post and ex-ante moral hazard and explore how these vary according to gender, age, education, and region, using a range of econometric techniques, including a novel ‘Blow Up and Cluster’ (BUC) method [3] which we do not believe has been used in this context previously. Our results are relevant for the general literature on health insurance and its impact on behaviours and actions but are particularly important for countries, such as Russia, who are reforming and modernising the structure and finance of their health care systems and debating the appropriate mix of social and private insurance.

Consistent with the early strands of related literature, we find compelling evidence of ex-post moral hazard but, in contrast to much of the empirical literature, we also find evidence of ex-ante moral hazard demonstrated by significant behavioural differences between those with self-funded and enterprise provided supplemental health insurance. We find limited evidence that either form of insurance is related to improved self-assessed health. We interpret these results as suggestive of the need for health insurance plans to incorporate more explicit incentives to ‘nudge’ behaviour in ways that will reduce the future flow of health care costs associated with risky health activities such as smoking, drinking and the absence of exercise. In addition, we note that further work needs to be undertaken to understand the efficiency-equity trade-offs implied by policies, such as mandatory health insurance, which increase access to health care while also stimulating its overuse.

We proceed as follows. Section two reviews the global literature on health insurance, health care and health related behaviour, making clear how important the institutional settings are for any empirical analysis. In section three, we therefore spell out the specificities of the Russian health care system and its financing. The subsequent section describes our data and details our empirical strategy, including as it relates to the crucial aspect of selection. Section five presents our empirical results which are then explored in greater detail in the final section, incorporating considerations for health policy as well as directions for future research.

## Health insurance, health care and health-related behaviour

The most straightforward theoretical implication of health insurance is that, other things being equal, it will result in increased consumption of health care due to what Arrow [4] famously identified as moral hazard. There are essentially two sources of moral hazard which can give rise to this phenomenon: ex-ante (relating to the individual propensity to invest in health) and ex-post (relating to the price sensitivity of demand for health care). The former, due to Ehrlich and Becker [5] captures the idea that health insurance reduces the incentives for individuals to invest in their health and, therefore, will be associated with unhealthy behaviours, including increased smoking and drinking, along with decreased exercise and less appropriate diets [6]. In contrast, ex-post moral hazard takes the individual’s health as given and posits that, at any given level of health, individuals with insurance will consume more health care, because the price of health care is lower [7].

While moral hazard provides for interesting theoretical implications, the theory does not provide us with predictions of the impact of health insurance on either health outcomes or on social welfare. First, the impact on health outcomes is unclear, because any (presumed) benefits of increased health care utilisation may be offset by ex-ante reductions in health investments. Second, diminishing marginal returns to health care imply that the impact of additional consumption depends on the initial individual stock of health capital [8]. Put differently, the impact on social welfare ultimately depends on the following combination of factors: the balance of increased (decreased) use of efficacious (inefficacious) medical services [9]; the relationship between health insurance and health behaviours; and the extent to which increased use of medical services is linked with positive changes in health behaviour.

To further complicate matters, identifying the existence of moral hazard presents a methodological challenge, since in most empirical settings, there are fundamental selection differences between those with and without insurance [10, 11]. The ‘high risks’ (i.e., least healthy) self-select into insurance, so that both adverse selection and moral hazard are consistent with greater ex-post utilisation of health care [12]. This renders it challenging to disentangle moral hazard from selection effects but is of crucial importance from a policy perspective, since policies to address adverse selection (e.g., mandatory insurance) may exacerbate moral hazard [13].

The classical theoretical studies referred to above paved the way for a rich seam of empirical health economics research, much of which has been linked to the famous health insurance ‘experiments’ implemented in the United States. Both the RAND Health Insurance Experiment, from the 1970s [1, 14] and the Oregon Health Insurance Experiment, from the 2000s [2] found compelling evidence of ex-post moral hazard: that is, spending on health care is lower when consumer cost-sharing and out-of-pocket spending requirements are higher. “Moral hazard, in other words, irrefutably exists” [15].

These findings have found succour outside of experimental settings, where additional care to avoid conflating moral hazard and adverse selection is called for. Inevitably, given the role of private and public insurance and the two experiments referred to above, much of this literature has focused on the United States. Expansions of Medicaid and Medicare, supplemental insurance (e.g., Medigap), and the Patient Protection and Affordable Care Act (ACA) reforms have all been strongly associated with increased utilisation of medical care with and without strong evidence of adverse selection [16–23].

European countries have had few policy ‘experiments’ comparable to those in the US and so the literature for European countries is sparser. France provides one interesting exception [24], with a natural co-payment experiment from the 1990s, confirming demand for some physician services is responsive to adjustments in the co-payment rate. The French system, not unlike the Russian system, offers patients more freedom to see a specialist directly, but, while insurance is shown to strongly influence overall use of physician services, there is no evidence of it proving decisive in the decision between physician and specialist [25]. Though not benefitting from the same natural exogeneity as in the French case, other institutional settings across Europe provide further evidence of ex-post moral hazard in Ireland [26], Denmark [27], Spain [28], Switzerland [29] and Germany [30].

Discussions of moral hazard have predominantly adopted the ex-post definition but, as the link between unhealthy behaviours, population health and the growth of health care expenditure becomes better understood, explorations of ex-ante moral hazard have proliferated, drawing attention to a related set of empirical challenges. There are myriad different—good and bad—health behaviours, including those associated with exercise, diet and the consumption of drugs and alcohol, all of which are influenced by multiple environmental, social and economic factors. Even where we observe that smoking or alcohol use do not increase after the uptake of insurance, it is not clear whether this is because the Grossman type theoretical incentives are inoperative (i.e., no pure ex-ante moral hazard) or whether the greater use of medical services has a counter-balancing effect on health behaviours [31, 32]. What evidence there is, is mixed.

From the RAND experiment [33], the authors find no empirical link between smoking or body weight and insurance coverage. Similarly, Finkelstein et al. [2] report no evidence of ex-ante moral hazard in the Oregon Medicaid experiment. In non-experimental settings, Dave and Kaestner [31] argue that Medicare is associated with lower levels of physical activity and increased smoking and drinking, while Courtemanche and Zapata [34] find that the Massachusetts reforms were associated with lower body mass index but did not affect smoking or physical activity. Following the recent ACA expansion of dependent insurance for young adults in the United States, Barbaresco et al. [35] find weak evidence of ex-ante moral hazard and only in the case of alcohol consumption patterns, while Courtemanche et al. [36], despite evidence of ex-post moral hazard, find limited impacts on either behaviours or outcomes.

More recently, Lee [37], examining the same reforms, finds that health insurance coverage has no effect on the health behaviours or preventative care of young Americans. Others argue [38] that the theoretical relationship between insurance and risky behaviours is ambiguous and find no empirical evidence that ACA Medicaid expansion resulted in increased risky health behaviours. In contrast, Stanciole [39], modelling the insurance and behaviour choices as sequential and interdependent, finds that health insurance has significant incentive effects on lifestyle choices, increasing the tendency towards heavy smoking, sedentarism and obesity and decreasing the tendency towards binge drinking. Kelly and Markowitz [40] conclude that health insurance can impact risky health behaviours, as well as outcomes and access, but that the effects are strongly influenced by the institutional context. Outside of the US, Courbage and Coulon [41] explore how private health insurance in the UK affects the demand for (insured) preventive care and (uninsured) individual behaviours. They find that health insurance increases the propensity for exercise and for breast screening while reducing the likelihood of smoking.

A crucial strand of the argument for extending coverage is that health insurance improves health [34] and yet, as with ex-ante moral hazard, the evidence linking health and health insurance is not conclusive. In the RAND experiment the positive impact of insurance on health is limited to certain sub-groups, such as the poor [14, 33]. Finkelstein et al. [2] find that self-reported physical and mental well-being both improve in the Oregon experiment, while expansions to Medicaid have been shown to improve infant health [20] and reduce child hospitalisations [21]. Evidence of the impact on health of Medicare is also mixed. Card et al. [18] find improved self-rated health for certain sub-groups, while Finkelstein and McKnight [42] find no evidence of improved mortality rates among the insured. Meanwhile, the Massachusetts reforms have been linked with improved self-assessed health, along with decreased mortality rates [38, 43], while the recent ACA dependent provision reforms have also been linked with improved self-reported health [36, 44]. A more recent strand of international literature, covering China, Japan and Canada [45–48] seeks to explore the possible causes of any potential positive health effect by identifying plausible mechanisms such as the link between increased interaction with health services and improved health behaviours.

In sum, while the fact of the relationship (if not the size) between insurance and ex-post moral hazard is both theoretically and empirically well-established, the link between insurance and preventative care use, risky health behaviours and health outcomes is both theoretically and empirically ambiguous. Health insurance has price and income effects as well as possible ex-ante moral hazard effects and the net outcomes are context and sub-group specific. More specifically, the effects of extending insurance coverage depend on the specific institutional features of the health-care system as well as the social, economic and demographic specificities which drive behaviour. We, therefore, turn next to the specifics of the Russian case.

## The Russian institutional context

The Russian health system is based on a mandatory health insurance (MHI) premium paid by employers. In principle, this finances a national health system which is free at the point of access. In reality, the health system is underfunded and inefficient and many Russians fail to access the health care they require. Indeed, drugs are not covered by Russian health insurance schemes, though there are complementary drug provision and subsidy schemes targeting sections of the population [49]. In practice, therefore, there are three (non-mutually exclusive) access routes to medical care in Russia: employer-financed MHI; out-of-pocket payments; or voluntary health insurance (VHI).^1^ As in the French system, discussed above, Russians have some discretion over whether to see a general practitioner or to go directly to a hospital-based specialist. The former is more common under the MHI system but, even then, there is regional variation and substantial flexibility.

A small minority of Russians have access to supplementary VHI schemes either through their employer (who gains tax benefits in return) or through purchasing them directly. The mandatory health insurance provides for a minimum defined set of free health care services, while VHI contracts expand the range of services available. These VHI schemes provide enhanced access to a higher quality range of health services which can be used in supplement to or instead of MHI and/or out-of-pocket payments. These two variants of health insurance exist simultaneously and often duplicate each other. Over time, it is hoped that the VHI schemes will replace what had become ubiquitous practices of, formal or less formal, out-of-pocket payments paving the way to more rapid or higher quality health care provision. For most Russians though, the cost of VHI is prohibitive and the self-financing of VHI contracts remains rare. Indeed, according to the Russian Bureau of Statistics [50], only 5.4% of all medical appointments are VHI-related and, in Moscow and St. Petersburg, corporate VHI contracts account for 95% of the VHI market. Nevertheless, the health insurance market is growing and the number of VHI contracts has increased from around 6.6 million in 2000 to almost 11.4 million in 2016, corresponding to approximately 4.5–8% of the Russian population [51]. More recent analysis shows that the turnover of the paid medical services market in Russia amounted to over 600 billion roubles in 2018, with market turnover growing at over 10% annually [52].

This set of institutional arrangements allows us to identify three sub-groups of the population: first, the largest group is reliant on MHI (supplemented where possible with out-of-pocket expenditure) only; second, is the group that receives supplemental VHI through association with their enterprise; and third, is the group that chooses to finance supplemental VHI themselves while still having access through MHI. Our interest is in observing how and whether membership of one or other of these insurance groupings impacts on health outcomes and health behaviours. These institutional configurations are helpful as they allow us to partially unpick some of the classical asymmetry of information challenges that characterise the relationship between health insurance, health outcomes and health behaviours. That is, although the three groups above are not mutually exclusive, we can identify two distinct ‘types’ of consumers—those that choose to purchase their own health insurance (as supplement to MHI) and those that have health insurance provided by their enterprises (as supplement to MHI). The price changes apply to both types but the mechanisms through which they are ‘selected’ for health insurance differ significantly. Therefore, while distinguishing either ex-post or ex-ante moral hazard from adverse selection is necessarily speculative in the case of those self-selecting into VHI, we argue that in the case of enterprise provided insurance, adverse selection is negligible, an argument that also receives support from US based studies [53]. Given the importance of this latter assumption, we discuss this ‘selection mechanism’ further in “Data and empirical methodology”.

Russia now finds itself at a crossroads. There is widespread recognition that the prevailing system of compulsory medical insurance is ineffective and that the share of private spending on health care, between 40 and 50%, is too high. The Covid-19 pandemic has further exposed some of the weaknesses in the system of financing. A number of policy proposals remain on the table, ranging from the replacement of the mandatory system with a purely voluntary system, the introduction of a co-payment system, the development of a comprehensive drug insurance system, or the further reduction of the mandatory offer alongside a more clearly differentiated expansion of the voluntary system. The research in this paper advances the understanding of where the inefficiencies lie, both in the mandatory and the voluntary health financing systems.

## Data and empirical methodology

### Data

This research draws on Phase II of the RLMS-HSE,^2^ which is a series of annual household surveys designed to monitor the health and economic welfare of households and individuals in Russia. Data has been collected each autumn since 1994 (other than in 1997 and 1999) and represents the only long-term, nationally representative, source of household and individual level data for the Russian Federation. In this paper we use the adult survey data from 2000 to 2017 inclusive (the years when information about VHI is included in the survey), restricting our sample to adults above mandatory schooling age (17) and up to age 72, beyond which labour market engagement is limited.^3^

The survey, which takes place each autumn, is based on the principle of ‘repeated sampling of dwellings’, in which all household members are interviewed in each survey (if they can be contacted within three visits), and then the dwelling itself (rather than the household) is followed over time. In combination with regular annual replenishments this sampling strategy maintains the cross-sectional representativeness of the sample for each round. Additionally, there is a component of the panel which is followed regardless of dwelling, and further attempts are also made to follow-up individuals who have moved out of the household. The round-by-round attrition, at a little under 10%, is not out of line with equivalent household panel surveys from elsewhere [54], while the 97% response rate of individuals within surveyed households is testament to the robustness of the survey protocol. The relative richness and reliability of these data allows us to draw on their longitudinal component to explore the relationship between VHI and health-related behaviours and outcomes, including as these are conditioned by important socio-economic and demographic heterogeneities.

We identify eight health outcome and behaviour (dependent) variables as well as a rich range of explanatory variables, including the VHI indicator. Full variable definitions are provided in Table 3. The first dependent variable, ‘visits to doctor’, is a variable with five categories, increasing sequentially from ‘less than annually’ through to ‘several times per month’ and represents a proxy for individual interaction with the health care system. This question has only featured in the RLMS-HSE survey since 2004, so the corresponding estimates refer to a slightly reduced sample. There are then 6 variables which proxy for individual health behaviours: smoking or not; quantity of cigarettes smoked among the sub-set of smokers; drinking or not; frequency of alcohol consumed among the sub-set of drinkers; regularity of physical exercise (question not included in the 2007 survey); and body mass index (BMI), disaggregated into deciles, as a proxy for health outcomes concerned with under- and overweight. Finally, self-assessed health (SAH) is a standard survey variable reflecting an individual’s assessment of their own overall health state on a 5-point scale, ranging from very bad to very good.

The main explanatory variable of interest is drawn from a question, included in the RLMS-HSE survey since 2000, asking if individuals have supplementary VHI. Respondents were subsequently asked whether they had paid for the VHI themselves, whether their enterprise/organisation had paid, or whether it was financed through some ‘other’ source. The share of VHI contracts paid by enterprises is approximately 80%, though higher in Moscow and St. Petersburg.^4^ In addition, we include a standard set of explanatory variables which are theoretically and empirically linked with health outcomes. At the individual level we control for log of monthly income measured in (Moscow, December 2006) roubles, the highest level of education achieved, and self-reported chronic illness (specifically heart, lung, kidney, liver, spinal or gastrointestinal). Income and education are traditional controls in so far as they determine access to and knowledge of medical services and healthy lifestyles. Table 4 demonstrates that the mean values of health indicators are different over respondents’ education levels, while Table 5 confirms, as expected, that chronic illness is a significant predictor of the dependent variables. At the household level, we follow the literature [39, 55, 56], in controlling for the presence of children aged under three, as a proxy for ‘young family’ status, and Table 5, shows that, in most cases, the mean values of healthy life indicators for people with children differ significantly from those without young children. At the macro level we control for year fixed effects to capture the influence of aggregate time trends.

Table 1 presents descriptive statistics for the dependent and explanatory variables according to whether the individual respondent reports having no VHI, having enterprise VHI (ever during 2000–2017 and concurrently with the response year), or having self-provided VHI (ever during 2000–2017 and concurrently with the response year). Those with enterprise VHI comprise of more males, are richer, report fewer chronic illnesses, have more young children and are more likely to be located in Moscow and St. Petersburg. They are more educated than those who never had VHI during the period but marginally less educated than those who are self-insured. Compared to those with no VHI, the latter are less male but richer, more educated and with more chronic illness reported. Turning to the eight health outcomes of interest, those without VHI have the lowest number of visits to the doctor, the lowest propensity to drink but also the lowest engagement with physical exercise and the lowest self-assessment of their health status. Comparing enterprise and self-provided VHI, visits to the doctor and physical exercise are lower among the former, but smoking, drinking and BMI are all higher. Self-assessed health is higher among those currently reporting VHI but lower among those ever having reported VHI.

**Table 1:** Descriptive statistics (mean values, standard deviations, number of observations, and number of respondents)

Variables (short notation in brackets)	Statistical notations	Without VHI from 2000 to 2017	Ever self, but not now	Ever enterprise, but not now	In years of self	In years of enterprise
(1) Visits to doctor (doctor)	Mean (S.d. between; S.d. within)	2.190 (0.880; 0.727)	2.254 (0.821; 0.774)	2.232 (0.761; 0.754)	2.527 (1.053; 0.404)	2.387 (0.903; 0.596)
	Observations; respondents	132,424; 28,336	5047; 801	15,510; 2785	867; 658	5877; 2827
(2) Smoker (smoke)	Mean (S.d. between; S.d. within)	0.346 (0.453; 0.188)	0.325 (0.415; 0.224)	0.370 (0.444; 0.214)	0.305 (0.463; 0.077)	0.374 (0.471; 0.163)
	Observations; respondents	159,430; 31,618	6102; 844	18,889; 2932	1030; 769	6615; 3177
(3) Cigarette consumption (smoke_n)	Mean (S.d. between; S.d. within)	15.450 (7.065; 4.535)	15.447 (7.097; 4.811)	15.383 (6.872; 4.662)	14.907 (7.639; 1.741)	15.714 (7.754; 3.090)
	Observations; respondents	54,402; 13,261	1960; 342	6939; 1384	311; 247	2451; 1296
(4) Drink (drink)	Mean (S.d. between; S.d. within)	0.710 (0.385; 0.288)	0.706 (0.373; 0.279)	0.790 (0.317; 0.276)	0.850 (0.380; 0.068)	0.840 (0.335; 0.197)
	Observations; respondents	121,704; 26,914	4593; 777	13,789; 2687	782; 596	5549; 2713
(5) Alcohol consumption (drink_n)	Mean (S.d. between; S.d. within)	2.417 (0.997; 0.736)	2.555 (0.971; 0.776)	2.548 (0.928; 0.774)	2.533 (1.158; 0.431)	2.584 (1.073; 0.594)
	Observations; respondents	84,789; 22,916	3538; 683	12,370; 2558	709; 518	4788; 2506
(6) Physical exercise (sport)	Mean (S.d. between; S.d. within)	1.518 (0.958; 0.809)	1.689 (0.903; 0.975)	1.607 (0.875; 0.878)	1.780 (1.246; 0.415)	1.733 (1.126; 0.730)
	Observations; respondents	149,416; 31,218	5723; 840	17,608; 2908	891; 667	6006; 2971
(7) BMI decile (bmi_dec)	Mean (S.d. between; S.d. within)	5.445 (2.692; 1.167)	5.510 (2.610; 1.259)	5.553 (2.669; 1.170)	5.415 (2.816; 0.456)	5.699 (2.768; 0.784)
	Observations; respondents	159,518; 31,621	6107; 844	18,903; 2932	1031; 770	6617; 3177
(8) SAH (sah)	Mean (S.d. between; S.d. within)	3.260 (0.629; 0.414)	3.368 (0.543; 0.439)	3.344 (0.486; 0.424)	3.310 (0.624; 0.166)	3.372 (0.573; 0.305)
	Observations; respondents	158,724; 31,576	6078; 844	18,818; 2931	1027; 766	6596; 3170
(9) Log (1 + Income)	Mean (S.d. between; S.d. within)	7.984 (2.683; 2.138)	8.256 (2.364; 2.322)	8.762 (1.827; 2.051)	8.781 (2.515; 0.767)	9.631 (1.613; 1.023)
	Observations; respondents	159,518; 31,621	6107; 844	18,903; 2932	1031; 770	6617; 3177
(10) Secondary school	Mean (S.d. between; S.d. within)	0.240 (0.444; 0.132)	0.153 (0.374; 0.154)	0.120 (0.308; 0.134)	0.138 (0.365; 0.031)	0.087 (0.301; 0.063)
	Observations; respondents	159,518; 31,621	6107; 844	18,903; 2932	1031; 770	6617; 3177
(11) Vocational training school	Mean (S.d. between; S.d. within)	0.224 (0.401; 0.068)	0.160 (0.338; 0.074)	0.200 (0.376; 0.085)	0.138 (0.350; 0.000)	0.163 (0.375; 0.046)
	Observations; respondents	159,518; 31,621	6107; 844	18,903; 2932	1031; 770	6617; 3177
(12) Technical college	Mean (S.d. between; S.d. within)	0.274 (0.417; 0.113)	0.244 (0.398; 0.140)	0.273 (0.420; 0.144)	0.229 (0.420; 0.062)	0.257 (0.432; 0.092)
	Observations; respondents	159,518; 31,621	6107; 844	18,903; 2932	1031; 770	6617; 3177
(13) University	Mean (S.d. between; S.d. within)	0.262 (0.422; 0.125)	0.443 (0.473; 0.170)	0.407 (0.473; 0.168)	0.496 (0.497; 0.062)	0.493 (0.493; 0.107)
	Observations; respondents	159,518; 31,621	6107; 844	18,903; 2932	1031; 770	6617; 3177
(14) Chronic disease	Mean (S.d. between; S.d. within)	0.389 (0.415; 0.298)	0.403 (0.400; 0.322)	0.373 (0.386; 0.319)	0.473 (0.486; 0.166)	0.372 (0.452; 0.231)
	Observations; respondents	159,518; 31,621	6107; 844	18,903; 2932	1031; 770	6617; 3177
(15) Children < 3 years in HH	Mean (S.d. between; S.d. within)	0.117 (0.267; 0.243)	0.120 (0.234; 0.264)	0.130 (0.252; 0.274)	0.099 (0.315; 0.093)	0.143 (0.308; 0.218)
	Observations; respondents	159,518; 31,621	6107; 844	18,903; 2932	1031; 770	6617; 3177
(16) = 1 if age < 36	Mean (S.d. between; S.d. within)	0.393 (0.481; 0.186)	0.442 (0.460; 0.238)	0.404 (0.455; 0.242)	0.415 (0.493; 0.115)	0.421 (0.485; 0.167)
	Observations; respondents	159,518; 31,621	6107; 844	18,903; 2932	1031; 770	6617; 3177
(17) Male	Mean (S.d. between; S.d. within)	0.423 (0.498; 0.000)	0.443 (0.497; 0.000)	0.497 (0.500; 0.000)	0.400 (0.496; 0.000)	0.523 (0.499; 0.000)
	Observations; respondents	159,518; 31,621	6107; 844	18,903; 2932	1031; 770	6617; 3177
(18) = 1 if low education (less than university)	Mean (S.d. between; S.d. within)	0.738 (0.422; 0.125)	0.557 (0.473; 0.170)	0.593 (0.473; 0.168)	0.504 (0.497; 0.062)	0.507 (0.493; 0.107)
	Observations; respondents	159,518; 31,621	6107; 844	18,903; 2932	1031; 770	6617; 3177
(19) = 1 if Moscow or St. Petersburg	Mean (S.d. between; S.d. within)	0.106 (0.342; 0.000)	0.103 (0.322; 0.000)	0.170 (0.413; 0.000)	0.136 (0.335; 0.000)	0.212 (0.421; 0.000)
	Observations; respondents	159,518; 31,621	6107; 844	18,903; 2932	1031; 770	6617; 3177

For variables (1)–(8) statistics refer to the corresponding number of non-missing values included in the estimates reported in Table 2 (male and female sample)

For variables (9)–(19), we use the (male and female) sample corresponding to that reported in Table 2 for “bmi_dec”

**Fig. 1:**
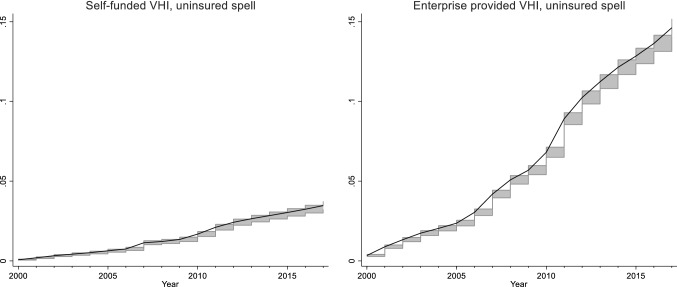
Nelson–Aalen cumulative hazard functions for uninsured stay, prior to self-funded and enterprise provided VHI (with pointwise 95% confidence bands)

**Fig. 2:**
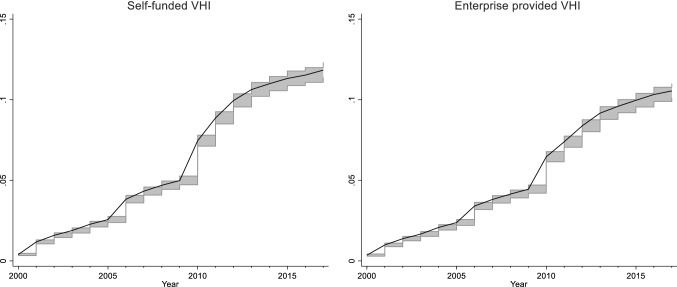
Nelson–Aalen cumulative hazard functions insured stay, for self-funded and enterprise provided VHI (with pointwise 95% confidence bands)

To further understand the data, in Figs. 1 and 2, we present cumulative hazard functions, taking into account that we have left truncation of VHI in our data. The hazard functions are estimated on the sample of observations for which we have non-missing values of the explanatory variables and which we use in our subsequent regression estimates. Figure 1 confirms that respondents have a greater chance of leaving an uninsured state for enterprise provided VHI rather than for self-funded VHI. Figure 2 suggests that there is slightly greater persistence in the enterprise funded case and that the differences become noticeable after 10 years. Table 6 illustrates that the sample churning (e.g., major sample changes in 2001, 2006, 2010 and 2014) and left and right censoring would render any single cumulative hazard curve (i.e., not considered in comparison) largely meaningless. However, for our purposes, where we compare the behaviour associated with two contract types, the long panel component of the RLMS-HSE data facilitates analysis in which we can control for time invariant individual unobservable characteristics using fixed effects (FE) models. Moreover, as Table 7 shows, insurance episodes tend to be of short duration (i.e., less than 3 years) which, combined with the churning evident in Table 6, means that we observe substantial within variation of the corresponding explanatory variables, allowing us to reliably estimate the correlation between insurance status and a range of potential moral hazard behaviours.

### Methods

The descriptive statistics, discussed above, begin to reveal a pattern across health outcomes and behaviours. However, evaluating impact effects based on the comparison of simple descriptive statistics overlooks the respondent specific characteristics, including environmental differences, which may be important drivers of health-related outcomes. To explore more systematically we use regression analysis, controlling for the observed confounding factors (income, education, chronic illness, children, year) described above. However, a further part of the between respondent variation in the outcome variables of interest could be due to unobserved confounders. To the extent that such factors are individual time-invariant we can control for any consequent bias using the deviations from the individual means of variables in the FE regression models. In the regression models, with various health indicators as dependent variables, $self$ and $ent$ are the key (binary) variables of interest. For this approach to be valid, we rely on the assumption that the trend behaviour across different categories is consistent across time, regardless of movement between categories. To guard against the possibility that this may not hold, as a robustness check, we repeat estimates for different samples, defined according to age, gender, education, region and, finally, without the respondents who were never insured in the period 2000–2017.

We estimate eight FE base regressions (corresponding to the eight dependent variables):

1 $$y_{it} = \alpha_{i} + \beta_{1} {\text{self}}_{it} + \beta_{2} {\text{ent}}_{it} + x^{\prime}_{it} \delta + \mu_{t} + \varepsilon_{it} ,$$

where $y_{it}$ is the response of individual $i$ in period $t$ to questions relating to the eight dependent variables; $x_{it} = (x_{1it} {\kern 1pt} x_{2it} \ldots x_{kit} )^{\prime}$ is the column-vector of the $k$ control variables (income, education, chronic illness, presence of children under 3 years in household); $\beta$s are the main parameters of interest (revealing the excess in the mean value of the dependent variable, $y$, for the corresponding insurance state in comparison with the uninsured state, holding all other control variables, time, and individual effects fixed); $\delta$ is a column-vector of parameters for the control variables; $\alpha_{i}$ is the respondents’ time invariant individual-specific unobservable characteristics (that could correlate with regressors); $\mu_{t}$ is time fixed effects; and $\varepsilon_{it}$ is the error term that captures unobservable individual and environmental characteristics that may vary between respondents and over years. We first estimate each base model regression for the full pooled sample, before then re-estimating for different sub-samples defined according to age, gender, education and region.

We then repeat this exercise in what amounts to an extensive series of robustness checks. First, we simply exclude from the sample those respondents who never had any form of VHI. Second, we estimate FE logit models for the binary dependent variables (‘smoke’ and ‘drink’):

2 $$\Pr (y_{it} = 1|{\text{self}}_{it} ,{\text{ent}}_{it} ,x_{it} ) = \Lambda (\alpha_{i} + \beta_{1} {\text{self}}_{it} + \beta_{2} {\text{ent}}_{it} + x^{\prime}_{it} \delta + \mu_{t} ),$$

where $\Lambda$ is the cumulative logistic distribution. Third, given the ordered choice nature of five of the dependent variables (doctor, drink_n, sport, bmi_dec, and sah), we might consider estimating the corresponding FE ordered choice logit:

3 $$\left\{ {\begin{array}{*{20}l} {\begin{array}{*{20}l} {\begin{array}{*{20}l} {y_{it}^{*} = \alpha_{i} + \beta_{1} {\text{self}}_{it} + \beta_{2} {\text{ent}}_{it} + x^{\prime}_{it} \delta + \mu_{t} + \varepsilon_{it} ,\quad i = 1,2, \ldots ,n,\quad t = 1,2, \ldots ,T,} \hfill \\ {y_{it} = j,\quad \gamma_{{i{\kern 1pt} j - 1}} < y_{it}^{*} \le \gamma_{ij} ,\quad j = 1,2, \ldots ,M - 1,\quad \gamma_{i0} = - \infty ,\quad \gamma_{iM} = \infty ,} \hfill \\ \end{array} } \hfill \\ \end{array} } \hfill \\ \end{array} } \right.$$

where $y_{it}^{*}$ in each model is one of the five latent variables which theoretically depend on the individual utility function values concerned with the respondent’s choice of the frequency of visits to doctor, alcohol consumption, level of physical activity, BMI decile, and self-assessed health;$y_{it}$ is the respondent’s answer to the corresponding ordered choice question or BMI decile; and $M$ is the number of possible responses from which respondents choose the $j$-th response (or else is mechanically located in the $j$-th BMI decile).

Unfortunately, in the case of the non-linear FE model (3), the $\hat{\beta }$ estimates derived from short panels are inconsistent due to the so-called ‘incidental parameters problem’ [57, 58], when estimating the $\gamma_{ij} - \alpha_{i}$ differences necessary to obtain the $\hat{\beta }$’s. Without being able to ‘naturally’ extend $T$ we attenuate the inconsistencies in our $\hat{\beta }$ estimates through the application of a ‘BUC’ (Blow up and Cluster) methodology. In this approach we replace each observation in the sample by $M - 1$ copies of itself (so-called ‘Blow-Ups’) and each of these $M - 1$ replications of the individual’s choice is dichotomised at a different cut-off point $\gamma_{ij}$. Crucially, this process of dichotomisation, which preserves as much information as possible concerning the changes in the dependent variable, means the BUC methodology is robust to finite samples [3] and is well suited to our data. The $\beta$ parameters, for each specification of Eq. (3), are estimated by the conditional logit model on the ‘blown up’ sample, clustering the standard errors according to the $i$ individuals and removing the need to estimate $\alpha_{i}$.

### The selection mechanism

At the core of this research lies the identifying assumption that there is no systematic selection mechanism at work in the way that individuals become associated with enterprise provided VHI. Given the pre-eminence of this assumption it merits further elaboration before we present our results, with respect to both the institutional framework prevailing in Russia and to the empirical stylised facts in the Russian data. As we have seen, accounting for just 5.4% of all medical appointments, VHI remains relatively niche in Russia. Most Russian citizens have little familiarity with insurance principles in general and very limited understanding of the benefits associated with VHI services. In part this reflects the absence of clear legislation separating the varieties of service available through VHI and MHI and in part, reflects the restrictions within VHI contracts themselves. Specifically, the contracts are limiting in terms of the range of services, the range of locations and the quantity of doctors and specialists, but most significantly, they do not cover the (significant) costs of prescribed medication.

From the companies’ perspective, there are substantive benefits from offering VHI contracts to employees. These include benefits (up to 6% of payroll budget) relating to salary payments, exemptions from social taxes on VHI contract payments, and exemptions from personal and value added tax on the premiums and benefits associated with VHI. Large corporations, particularly in Moscow and St. Petersburg—where the health care market is more developed—therefore, face strong incentives to offer VHI as part of the employment package. Notwithstanding these incentives, there is little to suggest that Russian job seekers place high value on the provision of supplementary health insurance. Recent surveys by two of Russia’s largest employment agencies, Headhunter and Superjob, confirm that salary, job prospects, job stability, work environment, colleagues, location and the nature of the work are all seen, by job seekers, as more important than any accompanying social package, which may include VHI.^5^

We consider that this restrictive institutional setting renders it very unlikely that enterprise provided VHI suffers from the kind of adverse selection that we may expect in more mature health markets. However, even for the US, the evidence suggests that adverse selection is unlikely to be a significant issue for employer-provided insurance [53]. Nevertheless, to reassure ourselves further about this, we examine the empirical regularities within the RLMS-HSE data for evidence of adverse selection.

Specifically, we construct five binary health indicators comprising of: self-assessed health (1 if SAH is average, good, or very good); health problems in the last 30 days; hospitalisation during the previous 3 months; operation during the last 12 months; whether the household faces financial constraints in obtaining treatment/medicine; and three (log) real income/expenditure variables: per capita household spending on medical services; per capita household spending on medicine; and total individual income received in the last 30 days. We then implement a series of *t* tests comparing the means of these indicators across two groups: those in employment who had no VHI in period *t* and changed their place of work during the subsequent year for employment in *t* + 1 that (1) included VHI and (2) did not include VHI. We then repeat this exercise for those who were not in employment (including secondary employment) in period *t*. The results (Tables 8, 9, 10, 11) reveal no systematic evidence of adverse selection into employer provided VHI. If anything, the results demonstrate that workers changing jobs are healthier and financially better equipped to undertake health expenditure.^6^

In sum, while we do not claim to have a perfect natural experiment, our understanding of the Russian institutional context and the stylised facts that we observe in the data provide for both strong arguments and empirical evidence that Russian workers do not choose their employer based on the expectation of receiving VHI. This being so, we are confident that we can distinguish the effects of post-contract opportunist behaviour from adverse selection and can compare pre- and post-contract behaviours and outcomes across groups. To this end, Russia follows France [24] in providing a rare opportunity to contribute robustly to a US-centric literature with a European example.

## Empirical results

Table 2 presents the $\beta_{1}$ and $\beta_{2}$ estimates and their ratios of separate regression estimates for the eight dependent variables, each, in turn, estimated on the total pooled sample and then on sub-samples defined by age (17–35; 36–72), gender (male; female), education (low; high) and region (Moscow/St. Petersburg; other regions). Tables 12, 13 and 14 present the corresponding equivalent estimates for our battery of robustness checks.

**Table 2:** $\beta_{1}$ and $\beta_{2}$ estimates and their ratios in FE models

	Doctor	Smoke	Smoke_n	Drink	Drink_n	Sport	bmi_dec	sah
Male and female								
$\hat{\beta }_{1}$	0.220*** (0.034)	− 0.012 (0.008)	0.275 (0.361)	0.061*** (0.014)	0.025 (0.041)	0.054 (0.038)	− 0.115* (0.048)	− 0.020 (0.017)
$\hat{\beta }_{2}$	0.143*** (0.015)	− 0.001 (0.004)	0.387** (0.149)	0.022*** (0.006)	0.045* (0.018)	0.045** (0.017)	0.058** (0.022)	− 0.006 (0.008)
$\hat{\beta }_{2} /\hat{\beta }_{1}$	0.650** (0.122)	0.074** (0.318)	1.405 (1.922)	0.360*** (0.132)	1.793 (2.997)	0.824 (0.652)	− 0.505 (0.286)	0.288 (0.462)
Observations	159,803	192,079	65,744	146,578	105,496	179,681	192,193	191,255
Respondents	32,486	35,970	15,251	30,935	26,688	35,547	35,974	35,929
Age 17–35								
$\hat{\beta }_{1}$	0.287*** (0.054)	− 0.033* (0.014)	0.168 (0.526)	0.069** (0.022)	0.029 (0.064)	0.002 (0.064)	− 0.173* (0.084)	− 0.041 (0.027)
$\hat{\beta }_{2}$	0.175*** (0.025)	− 0.004 (0.007)	0.174 (0.210)	0.024* (0.010)	0.041 (0.028)	0.063* (0.028)	0.090* (0.039)	− 0.015 (0.012)
$\hat{\beta }_{2} /\hat{\beta }_{1}$	0.608** (0.143)	0.120*** (0.209)	1.035 (3.466)	0.343*** (0.182)	1.421 (3.324)	27.420 (770.321)	− 0.518 (0.333)	0.370^+^ (0.391)
Observations	63,250	76,204	29,074	57,666	43,735	71,069	76,274	75,958
Respondents	16,539	18,645	8633	15,521	13,707	18,365	18,649	18,627
Age 36–72								
$\hat{\beta }_{1}$	0.189*** (0.044)	0.001 (0.009)	0.139 (0.500)	0.052** (0.019)	0.029 (0.054)	0.092 (0.047)	− 0.034 (0.059)	− 0.013 (0.022)
$\hat{\beta }_{2}$	0.114*** (0.020)	0.000 (0.004)	0.455* (0.215)	0.016* (0.008)	0.049* (0.025)	0.048* (0.021)	0.022 (0.027)	− 0.004 (0.010)
$\hat{\beta }_{2} /\hat{\beta }_{1}$	0.606* (0.179)	0.186 (4.247)	3.271 (11.863)	0.318*** (0.199)	1.699 (3.287)	0.523 (0.354)	− 0.653 (1.376)	0.306 (0.959)
Observations	96,553	115,875	36,670	88,912	61,761	108,612	115,919	115,297
Respondents	19,083	21,117	8303	18,256	15,649	20,924	21,117	21,090
Male								
$\hat{\beta }_{1}$	0.114* (0.050)	− 0.012 (0.015)	0.293 (0.468)	0.056** (0.021)	0.035 (0.066)	0.061 (0.061)	− 0.111 (0.081)	− 0.019 (0.027)
$\hat{\beta }_{2}$	0.156*** (0.020)	0.001 (0.006)	0.439* (0.183)	0.006 (0.008)	0.059* (0.026)	0.043 (0.024)	0.065* (0.033)	− 0.004 (0.011)
$\hat{\beta }_{2} /\hat{\beta }_{1}$	1.372 (0.635)	− 0.060 (0.486)	1.501 (2.477)	0.107*** (0.150)	1.670 (3.235)	0.717 (0.819)	− 0.592 (0.524)	0.190 (0.642)
Observations	68,931	83,274	47,562	63,384	54,086	77,888	83,311	82,876
Respondents	14,771	16,484	10,698	14,015	13,322	16,288	16,486	16,465
Female								
$\hat{\beta }_{1}$	0.290*** (0.045)	− 0.011 (0.009)	0.180 (0.510)	0.063*** (0.019)	0.016 (0.049)	0.049 (0.048)	− 0.118* (0.060)	− 0.021 (0.021)
$\hat{\beta }_{2}$	0.131*** (0.023)	− 0.004 (0.004)	0.138 (0.241)	0.038*** (0.009)	0.026 (0.025)	0.046 (0.024)	0.051 (0.030)	− 0.008 (0.011)
$\hat{\beta }_{2} /\hat{\beta }_{1}$	0.454*** (0.106)	0.317 (0.466)	0.764 (2.560)	0.598 (0.235)	1.565 (4.917)	0.949 (1.062)	− 0.430* (0.332)	0.376 (0.645)
Observations	90,872	108,805	18,182	83,194	51,410	101,793	108,882	108,379
Respondents	17,715	19,486	4553	16,920	13,366	19,259	19,488	19,464
Low education								
$\hat{\beta }_{1}$	0.173*** (0.049)	− 0.012 (0.011)	0.361 (0.479)	0.065** (0.021)	0.022 (0.061)	0.083 (0.051)	− 0.210** (0.071)	− 0.064** (0.024)
$\hat{\beta }_{2}$	0.119*** (0.022)	0.001 (0.005)	0.551** (0.190)	0.026** (0.009)	0.046 (0.025)	0.064** (0.022)	0.041 (0.031)	0.004 (0.011)
$\hat{\beta }_{2} /\hat{\beta }_{1}$	0.689 (0.232)	− 0.126 (0.452)	1.526 (2.089)	0.400** (0.188)	2.078 (5.807)	0.762 (0.536)	− 0.197*** (0.163)	− 0.069*** (0.171)
Observations	112,021	137,010	51,820	102,074	73,272	128,141	137,098	136,415
Respondents	24,202	27,315	12,273	22,814	19,603	26,965	27,319	27,283
High education								
$\hat{\beta }_{1}$	0.256*** (0.047)	− 0.011 (0.011)	0.004 (0.540)	0.053** (0.019)	0.028 (0.054)	0.025 (0.060)	− 0.014 (0.063)	0.019 (0.024)
$\hat{\beta }_{2}$	0.153*** (0.022)	− 0.003 (0.005)	0.259 (0.243)	0.021* (0.009)	0.062* (0.026)	0.016 (0.028)	0.064* (0.030)	− 0.023* (0.011)
$\hat{\beta }_{2} /\hat{\beta }_{1}$	0.596** (0.141)	0.257 (0.529)	63.487 (8399.449)	0.396** (0.221)	2.170 (4.233)	0.625 (1.833)	− 4.649 (21.430)	− 1.193 (1.592)
Observations	47,782	55,069	13,924	44,504	32,224	51,540	55,095	54,840
Respondents	10,044	10,762	3500	9701	8410	10,632	10,763	10,753
Moscow/St Petersburg								
$\hat{\beta }_{1}$	0.359*** (0.093)	0.029 (0.023)	0.377 (1.055)	0.026 (0.039)	0.128 (0.112)	0.215* (0.107)	0.148 (0.127)	0.075 (0.047)
$\hat{\beta }_{2}$	0.216*** (0.037)	0.003 (0.009)	0.351 (0.322)	0.016 (0.015)	0.094* (0.043)	0.112** (0.040)	0.028 (0.047)	− 0.005 (0.018)
$\hat{\beta }_{2} /\hat{\beta }_{1}$	0.603* (0.188)	0.088** (0.304)	0.930 (2.763)	0.614 (1.088)	0.733 (0.732)	0.521 (0.322)	0.188* (0.360)	− 0.071*** (0.237)
Observations	18,085	22,044	8854	16,219	12,743	20,400	22,052	21,929
Respondents	4456	5152	2388	4046	3937	5068	5154	5145
Other regions								
$\hat{\beta }_{1}$	0.193*** (0.036)	− 0.018* (0.008)	0.268 (0.385)	0.067*** (0.015)	0.008 (0.044)	0.028 (0.040)	− 0.156** (0.052)	− 0.034 (0.018)
$\hat{\beta }_{2}$	0.125*** (0.017)	− 0.002 (0.004)	0.406* (0.167)	0.024*** (0.007)	0.031 (0.020)	0.029 (0.018)	0.064** (0.025)	− 0.006 (0.009)
$\hat{\beta }_{2} /\hat{\beta }_{1}$	0.648* (0.150)	0.098*** (0.234)	1.515 (2.254)	0.352*** (0.130)	4.004 (22.550)	1.048 (1.680)	− 0.413** (0.211)	0.168** (0.273)
Observations	141,718	170,035	56,890	130,359	92,753	159,281	170,141	169,326
Respondents	28,030	30,818	12,863	26,889	22,751	30,479	30,820	30,784

Standard errors in parentheses. The significance tests reported for the $\hat{\beta }_{2} /\hat{\beta }_{1}$ rows tests that the absolute value of the ratio equals one. *p  <  0.05, **p  < 0.01, ***p  < 0.001. Variable names defined in Table 3

Our first main finding is that the provision or purchase of supplemental VHI in Russia is strongly associated with an increase in health care utilisation. In all cases, other than for males, the effect is statistically significantly larger in the case of self-provision than in the case of enterprise provision. For males, there is no significant difference in the magnitude of effect. In both cases, the effects are strongest among younger cohorts, females, those with more education and those in Moscow and St. Petersburg. In each sub-group, the within sub-group differential is greater for the self-insured compared to the enterprise insured. The effects of self-provision are much larger for women, which is consistent with the much greater access to health care that Russian females have. Overall, these results suggest that, although the self-insured effects appear to conflate adverse selection with moral hazard, in Russia—as in most other countries for which evidence exists—there is strong overall evidence of ex-post moral hazard.

Turning to ex-ante moral hazard, the results are less clear-cut, but no less interesting. The impact of supplemental insurance on whether an individual smokes or not is negligible. There is weak evidence overall that those with self-funded insurance are less likely to smoke and that this appears to be primarily driven by younger cohorts and those outside of Moscow and St. Petersburg. More interesting are the results for smoking intensity, where we find strong evidence of higher smoking for enterprise-insurance types, with particularly strong effects for males, older cohorts, the less educated, and those outside of Moscow and St. Petersburg. Given that smoking in Russia remains a highly gendered habit [59], these results are predominantly reflective of male, rather than female, health behaviours.

The results for alcohol consumption do not mirror those for smoking. Insurance, whether self- or enterprise-funded, is strongly positively associated with the propensity to drink. The size of the effect is once again significantly larger among those self-insuring, suggesting that there is an element of adverse selection in addition to ex-ante moral hazard. This pattern is consistent across all sub-groups though the effect is not present for either form of insurance in Moscow and St. Petersburg. When it comes to drinking intensity, the results are striking. There is no relationship between self-insurance and the consumption of alcohol among drinkers but there is a significant positive relationship between enterprise insurance and consumption among drinkers. In combination, the drinking results suggest that the positive impact of insurance on the likelihood of drinking is greater for those with self-insurance but that the impact on the amount consumed is only observed among those with enterprise VHI.

Levels of physical exercise are positively associated with enterprise VHI in aggregate and specifically for those with lower education and in Moscow and St. Petersburg. In the latter case though, there is a still stronger effect among the self-insured. Those with self-insurance also have lower BMI, a result which sustains for the youngest cohorts, for females, for the less educated and for those outside of Moscow and St. Petersburg. In contrast, those with enterprise level insurance tend towards a higher BMI decile, a result which also pertains to younger cohorts, males and those in other Russian regions and to the more highly educated.

Finally, turning to self-assessed health, the results are weaker, though we still detect some effects. For the less educated we find some evidence that those with self-insurance report lower health status, while for individuals with enterprise VHI, we find that only those with higher education report lower levels of health.

Tables 12, 13 and 14 present the robustness checks described in the previous section. Table 12, in which we exclude from the sample those respondents who never had any form of VHI, shows that the pattern of $\beta$ estimates, ratios and significant tests remains qualitatively similar to those reported in our main results. This reassures us that the impact of ex-ante moral hazard (if it is present among the insured respondents) is negligible. Similarly, the average marginal effects of regressors of interest, estimated under FE logit models, and their ratios, as well as the corresponding average marginal effects of BUC estimates, reveal no qualitative differences from our main results.

In the event that enterprise provided VHI does not cover all of the necessary medical expenses some respondents may also have self-provided VHI. Table 15 presents the number of these cases and so as a further robustness check, in Table 16, we repeat the estimates with these cases removed. A comparison of Table 16 with Table 2 shows that the results remain qualitatively the same, with the majority of $\beta$ estimates increasing in magnitude with the ‘purer’ sample. Self-purchased and workplace provided VHI could be partially funded by others (for example, via intra-family transfers in the case of self-provision, or through banks, or government ministries in the case of enterprise provision) and so, as a final robustness check, we confirm that the tenor of the results, excluding the observations detailed in Table 17, do not change.

**Fig. 3:**
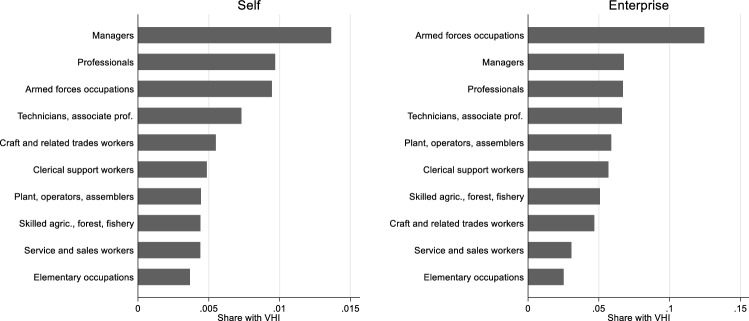
Share of VHI respondents in ISCO-08 occupations in the sample used for our regression models

**Fig. 4:**
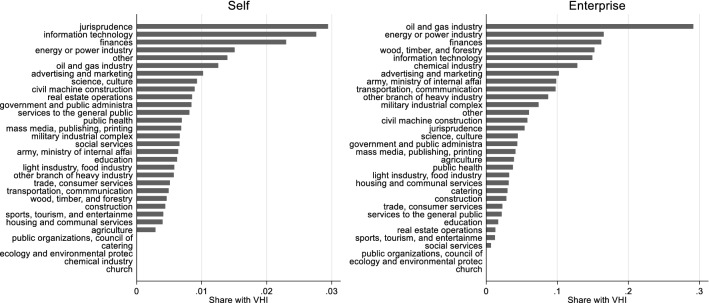
Share of VHI respondents over industries in the sample used for our regression models

Finally, it is likely that employer provided health insurance is non-uniform across industry and occupation. Figures 3 and 4 confirm that, broadly speaking, there are higher rates of VHI among managers, professionals and technical occupations in the IT, finance, energy and marketing and advertising sectors but that this is true regardless of whether self-provided or enterprise provided. Likewise, given that we are interested in the effects on those that actually have VHI, we don’t require uniformity of provision (or take up) of VHI, although—in thinking about policy implications—understanding of these distributions is instructive.

## Conclusions and discussion

The well-documented positive relationship between health insurance and health care utilisation has firmly established the existence of ex-post moral hazard as a necessary consideration to inform the design of health care systems and policies. In contrast, the theoretically and empirically ambiguous link between health insurance and preventative care use, risky health behaviours and health outcomes is a product of the specific institutional features of the health-care system and the social, economic and demographic characteristics within which it sits. Indeed, Harmon and Nolan [26] maintain that “the nature of demand for private health insurance itself depends on the institutional context in which that insurance operates”. This is an observation echoed in more recent studies discussing the mechanisms which may underpin the association between health insurance and health behaviours [45–48]. In this spirit, we offer the first empirical investigation of moral hazard in the health insurance sector in Russia. We provide robust evidence of ex-post moral hazard and, in contrast to some other literature, evidence also of ex-ante moral hazard.

Applying a range of econometric techniques, including a novel ‘Blow-Up and Cluster’ approach, to Russian longitudinal data, we compare the impact of enterprise provided and individually purchased supplemental VHI on eight health-related dependent variables proxying for ex-post and ex-ante moral hazard, in addition to self-assessed health. While adverse selection will likely distort the estimated impact of self-funded health insurance on behaviours and outcomes, we provide empirical and contextual arguments as to why this is unlikely to be the case with enterprise provided health insurance in Russia. Therefore, while we do not have an experimental setting, we do have two differing insurance ‘types’ who form their sub-groups through distinct selection mechanisms and provide us with a rare European setting in which we can disentangle moral hazard and adverse selection.

We find strong evidence that, as elsewhere, ex-post moral hazard should be an important consideration in designing health care policy. Post-contract visits to the doctor are unambiguously higher for individuals with supplemental health insurance. This is true in the pooled sample, in all sub-samples and is robust to different econometric specifications and approaches. Consistent with our expectations, the effects are larger for those who have self-insured but this likely reflects their self-selection (adverse selection) into health insurance plans. Equivalent adverse selection is difficult to contemplate in the case of enterprise provided health care, which is not a determining factor in Russian workers choosing their employer. We are, therefore, confident that, compared to those without supplemental insurance, Russian workers with enterprise provided insurance have engaged with the health care sector more regularly. In short, we concur with Einav and Finkelstein [15] that moral hazard, of the ex-post variety, “irrefutably exists”.

Consistent with the ambiguous theoretical predictions concerning ex-ante moral hazard our findings are less conclusive but are strongly suggestive of links between supplemental health insurance and ex-ante health behaviours and are more contingent on population demographics than our previous findings. The over and misuse of alcohol and tobacco, poor nutrition and diets, and a lack of physical exercise are notorious public health challenges in Russia and are major sources of mortality and morbidity, particularly among males. We find that those with self-insurance are less inclined to smoke than those with no insurance, they are more likely to be drinkers, but not to have higher consumption of alcohol, they have greater engagement with physical exercise and have lower BMI. In contrast, compared to those without insurance, individuals with enterprise VHI smoke more (particularly males, older cohorts, the less educated and those in the Russian regions), drink more (other than females) have higher BMI (other than older, more educated cohorts, in Moscow and St. Petersburg), and while their physical exercise is greater, it is less high than for those with self-provided VHI. In aggregate, we interpret these findings as strongly suggestive of an association between enterprise VHI and ex-ante moral hazard that is not observed among those who self-provide their VHI.

The net effect of insurance on health outcomes depends on the changes in both access to care and in health behaviours and, therefore, is theoretically ambiguous. The extent to which insurance-induced increases in health care utilisation translate to better health depends on the individual’s initial location along the health production function and on whether any changes in their health behaviours contribute to health improvements or, in the case of moral hazard, are linked with health deterioration. Our data offer us limited scope to explore health outcomes, beyond those that we control for in the regressions, but we find only limited evidence of a systematic relationship with self-assessed health (a reliable predictor of health outcomes). Ambiguity in these effects is consistent with findings from, for example, the RAND health insurance experiment and likely reflects that insurance can have positive effects for those that self-select into it (e.g., the chronically ill) but that these are offset by the negative impact that the need to self-select has in the first place.

In exploring whether and how these patterns vary according to population sub-group we are constrained somewhat by the number of observations, and our findings are, therefore, only suggestive. We do though discern clear patterns according to age, education, location and gender which are consistent with our understanding of the Russian context. In particular, health care infrastructure and insurance markets are more developed in Moscow and St. Petersburg, Russian women are much more likely to utilise health services and, in taking out health insurance, they have in mind their future engagement with health care services.

In Russia, as elsewhere, there is ongoing discussion concerning the role of supplemental insurance schemes aimed at expanding access to health care outside of the increasingly pressured core provision financed through mandatory health insurance. Our results make clear that insurance increases health care utilisation and increases unhealthy behaviours, albeit to different extents for different population sub-groups, but that it has no discernible impact upon immediate population health outcomes.

The tentative findings concerning ex-ante moral hazard are consistent with some of those emanating from the recent empirical literature from myriad jurisdictions, including the US [31, 39], Europe [60] and China [45, 47, 61]. The latter [61], finding strong evidence of ex-ante moral hazard in rural China, also provides a review of the literature from diverse developing countries which suggests that insurance-induced lifestyle changes impact indirectly on individual health through the behavioural changes induced via the provision of insurance. These changes in turn, may stem from differential access to health promotion and/or simply more regular contact with health professionals [31].

The finding that, separately from ex-post moral hazard, health insurance induces lifestyle changes that can damage individual health and undermine the public health system is self-evidently important in so far as it adds to the potential obstacles to achieving universal health coverage [62]. From a practical policy perspective, it suggests that the efficiency gains from understanding the effects of health insurance policies may be even greater than has been assumed. Indeed, understanding how co-payments and deductibles might lower health expenditure and reduce ex-post moral hazard is an empirical question facing all health insurance systems. However, there is growing evidence—including in this paper—that other policy tools (e.g., risk-rated premiums; or subsidised physician counselling and health promotion) targeting lifestyle behaviours could also increase efficiency and social welfare by attenuating distortions that health insurance introduces. In short, we have provided evidence that, in Russia—as elsewhere—insurance can shape behavioural impulses and so policy innovations targeting particular population sub-groups and behaviours such as excess consumption of tobacco and alcohol and incentivising improved nutrition and physical exercise will increase the efficiency of insurance policies and contribute to improved public health.

While we have contemplated the direction in which policy might look, this research also poses a series of questions that require further interrogation: Do the benefits of greater health care use outweigh the incentives to engage more freely in risky behaviours? Does enterprise provided insurance encourage those that under-use health care services (e.g., males, low income groups) to increase their utilisation and thereby positively address health inequalities? Why (in these data) is self-funded health insurance associated with reduced BMI but enterprise provided insurance linked with increased BMI? How can insurance plans simultaneously increase access to health care while also incentivising health behaviours that reduce the likelihood of health care being needed? As all countries confront the challenge of obtaining greater value from the resources they devote to health care, these questions and discerning credible answers to them become increasingly important. Far from being Russia-specific, our findings serve to emphasise the range of empirical questions that are pertinent to each contextual and institutional setting.

In addition to raising several crucial policy-related questions, several caveats to our analysis provide additional directions for future research. First, exploring a wider range of subjective, objective and clinical health outcomes is important for better understanding the ‘benefit’ side of supplemental health insurance. Second, examining changes in behaviour separately for those that do and do not already engage with the health system may provide further insight into the nature of moral hazard in Russian health care. Third, we take no account of expenditure or price data and thus are unable to disentangle income and price effects. Finally, collecting more nuanced insurance (e.g., across different contract types) and health data in Russia on a larger scale, particularly so that further sub-group analysis can be developed, will increase the power of the analysis and help to shed light on the mechanisms through which insurance and health-related behaviours are linked. This in turn will help us to understand where insurance expansion can be most beneficial and what kind of incentives should be built into its provision.

## Appendix

See Figs. 1, 2, 3 and 4 and Tables 3, 4, 5, 6, 7, 8, 9, 10, 11, 12, 13, 14, 15, 16 and 17.

**Table 3:** Variable definitions and their short notations (in brackets)

Variable name	Variable definition
Visits to doctor (doctor)	1, < annually; 2, annually; 3, 2–3 times a year; 4, monthly; 5, several times per month
Smoker (smoke)	1 if a respondent smokes, 0 otherwise
Cigarette consumption (smoke_n)	Number of cigarettes smoked per day by those who smoke
Drink (drink)	1 if a respondent ever drinks, 0 otherwise
Alcohol consumption (drink_n)	1, once last month; 2, 2–3 times last month; 3, weekly; 4, 2–3 times a week; 5, 4–6 times a week; 6, daily
Physical exercise (sport)	1, none; 2, light exercise; 3, moderate exercise; 4, intensive exercise; 5, daily
BMI decile (bmi_dec)	Body mass index as categorical variable representing deciles
SAH (sah)	Self-assessed health: 1, very bad; 2, bad; 3, average; 4, good; 5, very good
Log (1 + income)	The natural logarithm of respondent’s monthly income (+ 1) in Moscow Dec. 2006 rubles
Education	Defined by 4 dummy variables for: secondary school, vocational training school, technical college and university
Chronic disease	Self-reported heart, lung, kidney, liver, spinal or gastrointestinal disease
Children < 3 years in HH	1 if household has children under 3 years, 0 otherwise
Sub-samples grouped by	
= 1 if age < 36	1 if age below 36 years, 0 otherwise
Male	1 if male, 0 if female
= 1 if low education	1 if less than university education, 0 otherwise
= 1 if Moscow or St. Petersburg	1 if in Moscow or St. Petersburg, 0 otherwise

**Table 4:** Mean values of health indicators by education

Indicator	Secondary school	Vocational training school	Technical college	University	$\chi^{2}$
Visits to doctor (doctor)	2.154 (0.006)	2.065 (0.006)	2.240 (0.005)	2.289 (0.005)	1074.7***
Smoker (smoke)	0.336 (0.002)	0.518 (0.002)	0.312 (0.002)	0.257 (0.002)	7707.2***
Cigarette consumption (smoke_n)	15.576 (0.066)	16.463 (0.054)	14.835 (0.061)	14.521 (0.065)	655.8***
Drink (drink)	0.615 (0.002)	0.753 (0.002)	0.730 (0.002)	0.758 (0.002)	2054.8***
Alcohol consumption (drink_n)	2.451 (0.008)	2.624 (0.007)	2.336 (0.007)	2.415 (0.006)	866.4***
Physical exercise (sport)	1.598 (0.006)	1.341 (0.006)	1.467 (0.005)	1.732 (0.005)	3275.0***
BMI decile (bmi_dec)	5.493	5.448	5.620	5.335	285.7***
	(0.014)	(0.014)	(0.012)	(0.012)	
SAH (sah)	3.298 (0.003)	3.246 (0.003)	3.232 (0.003)	3.339 (0.003)	884.5***

Standard error in parentheses; ****p* < 0.001

**Table 5:** Mean values of health indicators by binary control variables

Indicator	Variable =	1 (yes)		0 (no)		*t* st
		Mean	St. err	Mean	St. err	
Visits to doctor (doctor)	Chronic disease	2.522	0.004	2.002	0.003	96.58***
	Children < 3 years in HH	2.173	0.008	2.203	0.003	− 3.71***
Smoker (smoke)	Chronic disease	0.321	0.002	0.361	0.001	− 18.64***
	Children < 3 years in HH	0.375	0.003	0.342	0.001	9.75***
Cigarette consumption (smoke_n)	Chronic disease	15.970	0.054	15.162	0.037	12.33***
	Children < 3 years in HH	15.153	0.083	15.496	0.033	− 3.86***
Drink (drink)	Chronic disease	0.710	0.002	0.725	0.001	− 6.19***
	Children < 3 years in HH	0.715	0.003	0.720	0.001	− 1.42
Alcohol consumption (drink_n)	Chronic disease	2.393	0.006	2.482	0.004	− 12.15***
	Children < 3 years in HH	2.418	0.010	2.454	0.004	− 3.42***
Physical exercise (sport)	Chronic disease	1.513	0.004	1.567	0.003	− 10.10***
	Children < 3 years in HH	1.483	0.007	1.555	0.003	− 9.20***
BMI decile (bmi_dec)	Chronic disease	5.480	0.011	5.466	0.008	1.09
	Children < 3 years in HH	5.668	0.019	5.445	0.007	11.14***
SAH (sah)	Chronic disease	2.906	0.002	3.516	0.002	− 211.81***
	Children < 3 years in HH	3.464	0.004	3.256	0.002	46.27***

****p* < 0.001

**Table 6:** Number of events in Nelson–Aalen cumulative hazard functions estimates (fail refers to end of spell)

Year	Self-funded VHI						Enterprise provided VHI					
	Uninsured spell (Table 4)			Insured spell (Table 5)			Uninsured spell (Table 4)			Insured spell (Table 5)		
	Fail	Lost	Enter	Fail	Lost	Enter	Fail	Lost	Enter	Fail	Lost	Enter
2000	22	6629	6026	115	5670	5089	102	102	102	104	5681	5096
2001	36	7236	6976	223	6110	5778	162	162	162	181	6158	5820
2002	39	7829	6986	116	6571	5826	129	129	129	109	6613	5864
2003	27	7624	7101	80	6422	5873	116	116	116	78	6461	5911
2004	26	7728	7083	103	6428	5781	83	83	83	109	6466	5811
2005	32	7377	7361	75	6081	6027	87	87	87	78	6102	6040
2006	31	9793	8945	323	8157	7348	182	182	182	263	8233	7412
2007	102	9609	8749	123	8020	7239	295	295	295	100	8100	7303
2008	20	9326	8588	86	7754	7075	220	220	220	79	7829	7133
2009	32	9245	8559	67	7686	7093	144	144	144	69	7742	7139
2010	80	14,301	12,087	547	12,320	10,237	259	259	259	447	12,470	10,358
2011	92	14,112	12,703	277	12,080	10,823	437	437	437	183	12,283	10,974
2012	63	14,703	12,746	194	12,695	10,890	253	253	253	179	12,852	11,016
2013	40	14,098	11,617	109	12,206	9947	168	168	168	128	12,320	10,044
2014	30	11,882	10,664	49	10,225	9110	131	131	131	58	10,321	9200
2015	28	11,822	10,834	41	10,212	9368	93	93	93	47	10,289	9430
2016	27	11,984	10,977	24	10,423	9569	97	97	97	42	10,476	9619
2017	28	12,165	0	33	10,672	0	111	111	111	24	10,740	0

**Table 7:** Cumulative years of each respondent insurance started and ended inside 2000–2017

Years	Self-funded VHI			Enterprise provided VHI		
	Frequency	Percent	Cumulative percent	Frequency	Percent	Cumulative percent
1	624	82.65	82.65	1708	61.95	61.95
2	76	10.07	92.72	508	18.43	80.38
3	28	3.71	96.42	252	9.14	89.52
4	14	1.85	98.28	130	4.72	94.23
5	1	0.13	98.41	57	2.07	96.30
6	7	0.93	99.34	37	1.34	97.64
7	3	0.40	99.74	26	0.94	98.59
8	1	0.13	99.87	16	0.58	99.17
9	1	0.13	100.00	8	0.29	99.46
10				6	0.22	99.67
11				6	0.22	99.89
12				2	0.07	99.96
13						
14				1	0.04	100.00
Respondents	755	100.00		2757	100.00

**Table 8:** Selection tests for working males (**p* < 0.05, ***p* < 0.01, ****p* < 0.001)

Indicator	New job + VHI		New job, noVHI		*t* st
	Mean	St. err	Mean	St. err	
SAH binary	0.992	0.008	0.965	0.007	2.45*
No health problems	0.776	0.037	0.722	0.018	1.29
Not hospitalized	1.000	0.000	0.977	0.006	3.78***
No operation	0.984	0.011	0.963	0.008	1.59
HH has money for treatment and medicine	0.783	0.046	0.803	0.019	− 0.40
Log (1 + Medical treatment per HH capita, 30 days)	6.289	0.320	6.046	0.117	0.71
Log (1 + Medicine bought per HH capita, 30 days)	5.182	0.110	5.002	0.055	1.47
Log (1 + Income)	8.950	0.239	8.770	0.098	0.70

**Table 9:** Selection tests for working females (**p* < 0.05, ***p* < 0.01, ****p* < 0.001)

Indicator	New job + VHI		New job, noVHI		*t* st
	Mean	St. err	Mean	St. err	
SAH binary	0.961	0.019	0.962	0.008	− 0.07
No health problems	0.574	0.049	0.643	0.021	− 1.29
Not hospitalized	0.941	0.023	0.966	0.008	− 1.01
No operation	0.941	0.023	0.957	0.009	− 0.62
HH has money for treatment and medicine	0.795	0.046	0.773	0.021	0.44
Log (1 + Medical treatment per HH capita, 30 days)	6.003	0.279	5.967	0.136	0.12
Log (1 + Medicine bought per HH capita, 30 days)	5.356	0.144	5.088	0.063	1.70
Log (1 + Income)	9.153	0.154	8.491	0.098	3.63***

**Table 10:** Selection tests for non-working males (**p* < 0.05, ***p* < 0.01, ****p* < 0.001)

Indicator	Job with VHI		Job without VHI		*t* st
	Mean	St. err	Mean	St. err	
SAH binary	0.938	0.035	0.965	0.009	− 0.75
No health problems	0.833	0.054	0.788	0.020	0.78
Not hospitalized	0.938	0.035	0.968	0.009	− 0.83
No operation	0.875	0.048	0.973	0.008	− 2.00
HH has money for treatment and medicine	0.865	0.057	0.793	0.023	1.17
Log (1 + Medical treatment per HH capita, 30 days)	5.891	0.420	5.780	0.156	0.25
Log (1 + Medicine bought per HH capita, 30 days)	4.754	0.233	4.867	0.083	− 0.46
Log (1 + Income)	4.050	0.634	3.899	0.206	0.23

**Table 11:** Selection tests for non-working females (**p* < 0.05, ***p* < 0.01, ****p* < 0.001)

Indicator	Job with VHI		Job without VHI		*t* st
	Mean	St. err	Mean	St. err	
SAH binary	0.962	0.026	0.940	0.010	0.80
No health problems	0.648	0.066	0.671	0.020	− 0.33
Not hospitalized	0.963	0.026	0.933	0.010	1.08
No operation	0.962	0.026	0.960	0.008	0.06
HH has money for treatment and medicine	0.707	0.072	0.756	0.020	− 0.65
Log (1 + Medical treatment per HH capita, 30 days)	6.128	0.476	6.210	0.144	− 0.16
Log (1 + Medicine bought per HH capita, 30 days)	5.127	0.209	5.121	0.062	0.03
Log (1 + Income)	4.305	0.578	4.310	0.165	− 0.01

**Table 12:** $\beta_{1}$, $\beta_{2}$, and their ratios in FE models estimated on sample of respondents who were voluntary insured for at least a year in 2000–2017

	Doctor	Smoke	Smoke_n	Drink	Drink_n	Sport	bmi_dec	sah
Male and female								
$\hat{\beta }_{1}$	0.211*** (0.034)	− 0.012 (0.009)	0.225 (0.359)	0.062*** (0.013)	0.025 (0.041)	0.059 (0.041)	− 0.112* (0.048)	− 0.019 (0.017)
$\hat{\beta }_{2}$	0.139*** (0.016)	− 0.001 (0.004)	0.321* (0.149)	0.023*** (0.006)	0.045* (0.018)	0.042* (0.018)	0.061** (0.022)	− 0.006 (0.008)
$\hat{\beta }_{2} /\hat{\beta }_{1}$	0.660** (0.131)	0.078** (0.346)	1.427 (2.367)	0.366*** (0.125)	1.819 (3.062)	0.715 (0.590)	− 0.543 (0.307)	0.331 (0.509)
Observations	27,379	32,649	11,342	24,874	20,707	30,265	32,675	32,531
Respondents	4150	4352	1990	4021	3772	4329	4353	4353
Age 17–35								
$\hat{\beta }_{1}$	0.288*** (0.055)	− 0.034* (0.015)	0.100 (0.517)	0.069** (0.021)	0.028 (0.065)	0.006 (0.069)	− 0.172* (0.082)	− 0.042 (0.027)
$\hat{\beta }_{2}$	0.172*** (0.025)	− 0.005 (0.007)	0.115 (0.209)	0.024* (0.010)	0.038 (0.029)	0.047 (0.031)	0.093* (0.038)	− 0.015 (0.013)
$\hat{\beta }_{2} /\hat{\beta }_{1}$	0.596** (0.144)	0.137*** (0.220)	1.143 (6.236)	0.345*** (0.176)	1.369 (3.353)	7.886 (91.976)	− 0.543 (0.342)	0.358 (0.381)
Observations	11,107	13,602	5057	9901	8801	12,556	13,616	13,557
Respondents	2226	2439	1198	2096	2077	2424	2440	2440
Age 36–72								
$\hat{\beta }_{1}$	0.172*** (0.045)	0.001 (0.010)	0.130 (0.497)	0.053** (0.018)	0.029 (0.054)	0.092 (0.051)	− 0.028 (0.058)	− 0.009 (0.022)
$\hat{\beta }_{2}$	0.104*** (0.021)	0.000 (0.005)	0.386 (0.215)	0.018* (0.008)	0.053* (0.025)	0.046* (0.023)	0.022 (0.027)	− 0.004 (0.010)
$\hat{\beta }_{2} /\hat{\beta }_{1}$	0.607* (0.200)	0.370 (9.937)	2.960 (11.397)	0.341*** (0.185)	1.853 (3.587)	0.495 (0.373)	− 0.790 (1.888)	0.422 (1.470)
Observations	16,272	19,047	6285	14,973	11,906	17,709	19,059	18,974
Respondents	2625	2749	1156	2552	2346	2726	2749	2749
Male								
$\hat{\beta }_{1}$	0.104* (0.052)	− 0.012 (0.016)	0.228 (0.463)	0.055** (0.020)	0.036 (0.066)	0.063 (0.066)	− 0.104 (0.078)	− 0.019 (0.027)
$\hat{\beta }_{2}$	0.149*** (0.021)	0.000 (0.006)	0.356 (0.182)	0.006 (0.008)	0.058* (0.026)	0.036 (0.026)	0.058 (0.032)	− 0.002 (0.011)
$\hat{\beta }_{2} /\hat{\beta }_{1}$	1.435 (0.746)	− 0.003 (0.521)	1.559 (3.247)	0.106*** (0.146)	1.616 (3.037)	0.564 (0.724)	− 0.557 (0.522)	0.097 (0.598)
Observations	13,220	15,764	8333	11,988	11,288	14,588	15,773	15,694
Respondents	2094	2195	1410	2029	1990	2182	2196	2196
Female								
$\hat{\beta }_{1}$	0.281*** (0.046)	− 0.010 (0.010)	0.158 (0.514)	0.066*** (0.018)	0.016 (0.050)	0.057 (0.052)	− 0.120* (0.061)	− 0.019 (0.022)
$\hat{\beta }_{2}$	0.130*** (0.024)	− 0.003 (0.005)	0.099 (0.246)	0.039*** (0.009)	0.026 (0.025)	0.049 (0.026)	0.065* (0.031)	− 0.010 (0.011)
$\hat{\beta }_{2} /\hat{\beta }_{1}$	0.462*** (0.113)	0.295 (0.553)	0.625 (2.573)	0.592 (0.216)	1.658 (5.481)	0.872 (0.922)	− 0.543 (0.375)	0.551 (0.859)
Observations	14,159	16,885	3009	12,886	9419	15,677	16,902	16,837
Respondents	2056	2157	580	1992	1782	2147	2157	2157
Low education								
$\hat{\beta }_{1}$	0.160** (0.051)	− 0.012 (0.013)	0.325 (0.485)	0.065*** (0.020)	0.028 (0.061)	0.091 (0.056)	− 0.203** (0.070)	− 0.062* (0.024)
$\hat{\beta }_{2}$	0.114*** (0.022)	0.002 (0.006)	0.449* (0.194)	0.025** (0.008)	0.049 (0.026)	0.059* (0.024)	0.037 (0.031)	0.004 (0.011)
$\hat{\beta }_{2} /\hat{\beta }_{1}$	0.711 (0.262)	− 0.127 (0.478)	1.384 (2.149)	0.393*** (0.175)	1.746 (3.952)	0.644 (0.480)	− 0.184*** (0.167)	− 0.059*** (0.176)
Observations	15,772	19,319	7562	14,138	11,883	17,886	19,334	19,243
Respondents	2585	2825	1381	2452	2339	2811	2826	2826
High education								
$\hat{\beta }_{1}$	0.246*** (0.048)	− 0.011 (0.012)	− 0.017 (0.523)	0.054** (0.018)	0.022 (0.055)	0.026 (0.061)	− 0.016 (0.064)	0.021 (0.024)
$\hat{\beta }_{2}$	0.151*** (0.023)	− 0.002 (0.006)	0.223 (0.238)	0.022* (0.009)	0.057* (0.026)	0.019 (0.029)	0.069* (0.031)	− 0.023* (0.011)
$\hat{\beta }_{2} /\hat{\beta }_{1}$	0.614* (0.151)	0.200 (0.569)	− 12.846 (387.470)	0.403** (0.212)	2.638 (6.805)	0.701 (1.958)	− 4.283 (17.233)	− 1.082 (1.323)
Observations	11,607	13,330	3780	10,736	8824	12,379	13,341	13,288
Respondents	1971	2038	764	1922	1804	2021	2039	2039
Moscow/St Petersburg								
$\hat{\beta }_{1}$	0.359*** (0.094)	0.033 (0.025)	0.346 (1.051)	0.026 (0.037)	0.105 (0.110)	0.221* (0.107)	0.160 (0.121)	0.071 (0.046)
$\hat{\beta }_{2}$	0.218*** (0.038)	0.003 (0.009)	0.393 (0.325)	0.017 (0.014)	0.091* (0.043)	0.118** (0.040)	0.045 (0.045)	− 0.008 (0.017)
$\hat{\beta }_{2} /\hat{\beta }_{1}$	0.608* (0.193)	0.091** (0.299)	1.136 (3.604)	0.666 (1.087)	0.870 (1.008)	0.534 + (0.318)	0.281* (0.359)	− 0.116*** (0.251)
Observations	4292	5194	1926	3830	3461	4785	5196	5172
Respondents	790	865	418	740	777	861	866	866
Other regions								
$\hat{\beta }_{1}$	0.180*** (0.037)	− 0.019* (0.009)	0.228 (0.383)	0.068*** (0.014)	0.010 (0.044)	0.031 (0.045)	− 0.151** (0.053)	− 0.033 (0.018)
$\hat{\beta }_{2}$	0.120*** (0.017)	− 0.002 (0.005)	0.321 (0.168)	0.024*** (0.007)	0.032 (0.020)	0.026 (0.021)	0.065* (0.025)	− 0.006 (0.009)
$\hat{\beta }_{2} /\hat{\beta }_{1}$	0.665* (0.166)	0.107*** (0.248)	1.407 (2.460)	0.355*** (0.123)	3.326 (15.273)	0.837 (1.381)	− 0.430* (0.225)	0.185** (0.283)
Observations	23,087	27,455	9416	21,044	17,246	25,480	27,479	27,359
Respondents	3360	3487	1572	3281	2995	3468	3487	3487

Standard errors in parentheses. The significance tests reported for the $\hat{\beta }_{2} /\hat{\beta }_{1}$ rows tests that the absolute value of the ratio equals one

**p* < 0.05, ***p* < 0.01, ****p* < 0.001

**Table 13:** Average marginal effects of $self$ and $ent$ conditional on zero individual fixed effects, $\alpha_{i}$, and their ratios in FE logit models

	Smoke	Drink
Male and female		
Self	− 0.046 (0.033)	0.209*** (0.041)
Ent	− 0.001 (0.014)	0.054*** (0.016)
Self/ent	0.018** (0.313)	0.259*** (0.090)
Observations	42,299	67,017
Respondents	5231	9781
Age 17–35		
Self	− 0.089* (0.040)	0.224*** (0.064)
Ent	− 0.012 (0.018)	0.049* (0.024)
Self/ent	0.131*** (0.207)	0.219*** (0.123)
Observations	19,419	23,804
Respondents	2924	4124
Age 36–72		
Self	0.013 (0.054)	0.184*** (0.056)
Ent	0.001 (0.021)	0.045* (0.021)
Self/ent	0.091 (1.723)	0.244*** (0.126)
Observations	19,459	40,126
Respondents	2433	5837
Male		
Self	− 0.056 (0.046)	0.206** (0.072)
Ent	0.001 (0.019)	0.010 (0.024)
Self/ent	− 0.010** (0.339)	0.050*** (0.116)
Observations	25,400	24,160
Respondents	3131	3616
Female		
Self	− 0.043 (0.043)	0.210*** (0.050)
Ent	− 0.013 (0.020)	0.084*** (0.020)
Self/ent	0.306 (0.579)	0.398*** (0.134)
Observations	16,899	42,857
Respondents	2100	6165
Low education		
Self	− 0.054 (0.045)	0.231*** (0.061)
Ent	0.003 (0.019)	0.068** (0.021)
Self/ent	− 0.055** (0.352)	0.293*** (0.119)
Observations	30,027	45,791
Respondents	3859	6919
High education		
Self	− 0.052 (0.054)	0.156** (0.051)
Ent	− 0.001 (0.025)	0.043* (0.021)
Self/ent	0.017* (0.489)	0.279*** (0.162)
Observations	10,465	19,365
Respondents	1396	2946
Moscow/St Petersburg		
Self	0.113 (0.099)	0.125 (0.114)
Ent	0.014 (0.034)	0.043 (0.035)
Self/ent	0.123** (0.325)	0.345 (0.414)
Observations	4914	7343
Respondents	719	1217
Other regions		
Self	− 0.071* (0.036)	0.218*** (0.043)
Ent	− 0.005 (0.016)	0.058*** (0.017)
Self/ent	0.064*** (0.228)	0.265*** (0.095)
Observations	37,385	59,674
Respondents	4512	8564

Standard errors in parentheses. The significance tests reported for the $\hat{\beta }_{2} /\hat{\beta }_{1}$ rows tests that the marginal effects ratio equals one. **p*  < 0.05, ***p*  <  0.01, ****p*  < 0.001

**Table 14:** Average marginal effects of $self$ and $ent$ conditional on zero individual fixed effects, $\alpha_{i}$, and their ratios in BUC models

	Doctor	Drink_n	Sport	bmi_dec	sah
Male and female					
Self	0.128*** (0.022)	0.014 (0.023)	0.032 (0.024)	− 0.047* (0.022)	− 0.021 (0.021)
Ent	0.085*** (0.010)	0.024* (0.010)	0.024* (0.011)	0.025* (0.010)	− 0.007 (0.009)
Self/ent	0.661* (0.134)	1.653 (2.693)	0.737 (0.650)	− 0.543 (0.333)	0.323 (0.552)
Blown up observations	282,274	188,461	280,435	559,261	175,824
Respondents	20,727	15,344	14,825	23,472	18,304
Age 17–35					
Self	0.157*** (0.032)	0.015 (0.031)	0.003 (0.028)	− 0.064 (0.033)	− 0.046 (0.032)
Ent	0.097*** (0.014)	0.019 (0.013)	0.026* (0.013)	0.034* (0.015)	− 0.017 (0.014)
Self/ent	0.618* (0.156)	1.276 (2.758)	7.541 (61.655)	− 0.541 (0.371)	0.366 (0.414)
Blown up observations	102,759	68,481	112,747	222,011	61,803
Respondents	9460	7257	7863	11,294	8400
Age 36–72					
Self	0.068** (0.021)	0.016 (0.032)	0.059 (0.035)	− 0.012 (0.026)	− 0.010 (0.021)
Ent	0.041*** (0.011)	0.027 (0.014)	0.028 (0.015)	0.009 (0.012)	− 0.004 (0.010)
Self/ent	0.608* (0.191)	1.675 (3.495)	0.467 (0.371)	− 0.754 (1.830)	0.386 (1.227)
Blown up observations	166,320	108,528	149,180	302,140	104,057
Respondents	12,431	8998	7610	13,895	10,872
Male					
Self	0.071* (0.030)	0.018 (0.033)	0.032 (0.034)	− 0.042 (0.033)	− 0.008 (0.030)
Ent	0.095*** (0.013)	0.028* (0.012)	0.020 (0.013)	0.028* (0.014)	− 0.005 (0.012)
Self/ent	1.337 (0.585)	1.540 (2.907)	0.614 (0.751)	− 0.651 (0.610)	0.600 (2.688)
Blown up observations	109,063	108,522	121,800	255,134	77,927
Respondents	8949	8145	6673	10,685	8438
Female					
Self	0.161*** (0.029)	0.009 (0.031)	0.030 (0.033)	− 0.049 (0.029)	− 0.031 (0.028)
Ent	0.074*** (0.014)	0.015 (0.016)	0.026 (0.017)	0.023 (0.015)	− 0.009 (0.014)
Self/ent	0.458*** (0.120)	1.661 (5.803)	0.878 (1.144)	− 0.462 (0.401)	0.288 (0.522)
Blown up observations	173,211	79,939	158,635	304,127	97,897
Respondents	11,778	7199	8152	12,787	9866
Low education					
Self	0.098** (0.031)	0.013 (0.032)	0.055 (0.038)	− 0.084** (0.031)	− 0.066* (0.030)
Ent	0.071*** (0.014)	0.023 (0.012)	0.042* (0.017)	0.019 (0.014)	0.005 (0.013)
Self/ent	0.727 (0.263)	1.817 (4.745)	0.761 (0.609)	− 0.222*** (0.187)	− 0.079*** (0.193)
Blown up observations	191,061	129,917	173,897	397,562	123,655
Respondents	14,774	10,903	9991	17,389	13,414
High education					
Self	0.147*** (0.030)	0.017 (0.035)	0.017 (0.037)	− 0.004 (0.031)	0.023 (0.029)
Ent	0.089*** (0.014)	0.036* (0.016)	0.006 (0.017)	0.028 (0.015)	− 0.027 (0.014)
Self/ent	0.604* (0.156)	2.124 (4.420)	0.362 (1.277)	− 6.681 (49.719)	− 1.194 (1.614)
Blown up observations	84,041	53,687	95,363	142,101	47,640
Respondents	6531	4745	5326	6961	5327
Moscow/St Petersburg					
Self	0.196** (0.060)	0.066 (0.056)	0.097 (0.057)	0.079 (0.054)	0.095 (0.060)
Ent	0.115*** (0.022)	0.049* (0.023)	0.058** (0.022)	0.013 (0.022)	− 0.006 (0.021)
Self/ent	0.586 (0.218)	0.739 (0.714)	0.604 (0.430)	0.159** (0.304)	− 0.062*** (0.226)
Blown up observations	31,469	22,071	33,507	55,097	18,542
Respondents	2588	2083	2212	3033	2345
Other regions					
Self	0.114*** (0.023)	0.005 (0.025)	0.019 (0.027)	− 0.063** (0.023)	− 0.037 (0.022)
Ent	0.076*** (0.011)	0.017 (0.010)	0.015 (0.012)	0.028* (0.011)	− 0.007 (0.010)
Self/ent	0.668* (0.162)	3.446 (17.823)	0.773 (1.270)	− 0.439* (0.243)	0.184** (0.293)
Blown up observations	250,805	166,390	246,928	504,164	157,282
Respondents	18,139	13,261	12,613	20,439	15,959

Standard errors in parentheses. The significance tests reported for the $\hat{\beta }_{2} /\hat{\beta }_{1}$ rows tests that the marginal effects ratio equals one

**p* < 0.05, ***p* < 0.01, ****p* < 0.001

**Table 15:** Two-way tabulation of self-purchased and workplace provided VHI

			Ent		Total
			0	1	
Doctor	Self	0	153,188	5748	158,936
		1	738	129	867
		Total	153,926	5877	159,803
Smoke	Self	0	184,578	6471	191,049
		1	886	144	1,030
		Total	185,464	6615	192,079
Smoke_n	Self	0	63,030	2403	65,433
		1	263	48	311
		Total	63,293	2451	65,744
Drink	Self	0	140,368	5428	145,796
		1	661	121	782
		Total	141,029	5549	146,578
Drink_n	Self	0	100,112	4675	104,787
		1	596	113	709
		Total	100,708	4788	105,496
Sport	Self	0	172,912	5878	178,790
		1	763	128	891
		Total	173,675	6006	179,681
bmi_dec	Self	0	184,689	6473	191,162
		1	887	144	1031
		Total	185,576	6617	192,193
sah	Self	0	183,776	6452	190,228
		1	883	144	1027
		Total	184,659	6596	191,255

**Table 16:** $\beta_{1}$ and $\beta_{2}$ estimates and their ratios in FE models without years when respondent has both self-purchased and workplace provided VHI

	Doctor	Smoke	Smoke_n	Drink	Drink_n	Sport	bmi_dec	sah
Male and female								
$\hat{\beta }_{1}$	0.245*** (0.036)	− 0.016 (0.009)	0.472 (0.391)	0.077*** (0.015)	− 0.000 (0.044)	0.075 (0.041)	− 0.140** (0.052)	− 0.016 (0.018)
$\hat{\beta }_{2}$	0.148*** (0.015)	− 0.001 (0.004)	0.417** (0.150)	0.024*** (0.006)	0.041* (0.018)	0.049** (0.017)	0.055* (0.022)	− 0.005 (0.008)
$\hat{\beta }_{2} /\hat{\beta }_{1}$	0.604*** (0.108)	0.064*** (0.233)	0.885 (0.785)	0.317*** (0.102)	− 176.386 (3.4e+04)	0.649 (0.412)	− 0.395** (0.222)	0.306 (0.594)
Observations	159,674	191,935	65,696	146,457	105,383	179,553	192,049	191,111
Respondents	32,480	35,962	15,245	30,928	26,680	35,539	35,966	35,921
Age 17–35								
$\hat{\beta }_{1}$	0.331*** (0.058)	− 0.037* (0.015)	0.314 (0.569)	0.081*** (0.024)	0.045 (0.069)	0.032 (0.070)	− 0.214* (0.090)	− 0.035 (0.029)
$\hat{\beta }_{2}$	0.182*** (0.025)	− 0.004 (0.007)	0.188 (0.212)	0.026* (0.010)	0.042 (0.028)	0.067* (0.029)	0.084* (0.039)	− 0.014 (0.012)
$\hat{\beta }_{2} /\hat{\beta }_{1}$	0.550*** (0.120)	0.120*** (0.187)	0.597 (1.249)	0.316*** (0.151)	0.950 (1.580)	2.103 (4.656)	− 0.391* (0.250)	0.394 (0.471)
Observations	63,201	76,149	29,057	57,619	43,697	71,019	76,219	75,903
Respondents	16,537	18,643	8632	15,519	13,703	18,363	18,647	18,625
Age 36–72								
$\hat{\beta }_{1}$	0.200*** (0.048)	− 0.002 (0.010)	0.437 (0.540)	0.070*** (0.020)	− 0.035 (0.059)	0.113* (0.051)	− 0.048 (0.064)	− 0.006 (0.024)
$\hat{\beta }_{2}$	0.116*** (0.020)	0.001 (0.004)	0.489* (0.217)	0.019* (0.008)	0.040 (0.025)	0.051* (0.021)	0.022 (0.027)	− 0.003 (0.010)
$\hat{\beta }_{2} /\hat{\beta }_{1}$	0.582* (0.170)	− 0.310 (3.011)	1.118 (1.446)	0.275*** (0.142)	− 1.124 (2.037)	0.454* (0.274)	− 0.458 (0.845)	0.480 (2.570)
Observations	96,473	115,786	36,639	88,838	61,686	108,534	115,830	115,208
Respondents	19,076	21,108	8297	18,248	15,641	20,915	21,108	21,081
Male								
$\hat{\beta }_{1}$	0.161** (0.055)	− 0.023 (0.016)	0.515 (0.509)	0.065** (0.023)	0.008 (0.073)	0.063 (0.067)	− 0.155 (0.088)	− 0.025 (0.030)
$\hat{\beta }_{2}$	0.161*** (0.020)	0.000 (0.006)	0.466* (0.184)	0.007 (0.008)	0.056* (0.026)	0.044 (0.024)	0.061 (0.033)	− 0.004 (0.011)
$\hat{\beta }_{2} /\hat{\beta }_{1}$	1.005 (0.363)	– 0.018*** (0.268)	0.906 (0.948)	0.106*** (0.127)	7.164 (66.558)	0.704 (0.821)	− 0.393 (0.317)	0.179 (0.496)
Observations	68,875	83,208	47,531	63,331	54,031	77,828	83,245	82,810
Respondents	14,766	16,477	10,694	14,009	13,315	16,281	16,479	16,458
Female								
$\hat{\beta }_{1}$	0.297*** (0.048)	− 0.012 (0.009)	0.317 (0.547)	0.083*** (0.020)	− 0.007 (0.053)	0.080 (0.052)	− 0.131* (0.064)	− 0.011 (0.023)
$\hat{\beta }_{2}$	0.135*** (0.023)	− 0.004 (0.004)	0.177 (0.244)	0.042*** (0.010)	0.020 (0.025)	0.053* (0.024)	0.050 (0.031)	− 0.005 (0.011)
$\hat{\beta }_{2} /\hat{\beta }_{1}$	0.455*** (0.105)	0.300 (0.434)	0.559 (1.209)	0.502** (0.166)	− 2.662 (19.534)	0.658 (0.507)	− 0.381* (0.303)	0.494 (1.418)
Observations	90,799	108,727	18,165	83,126	51,352	101,725	108,804	108,301
Respondents	17,714	19,485	4551	16,919	13,365	19,258	19,487	19,463
Low education								
$\hat{\beta }_{1}$	0.217*** (0.052)	− 0.017 (0.012)	0.465 (0.517)	0.072** (0.022)	− 0.038 (0.065)	0.095 (0.054)	− 0.245*** (0.074)	− 0.068** (0.025)
$\hat{\beta }_{2}$	0.125*** (0.022)	0.001 (0.005)	0.565** (0.191)	0.027** (0.009)	0.038 (0.026)	0.065** (0.022)	0.037 (0.032)	0.004 (0.011)
$\hat{\beta }_{2} /\hat{\beta }_{1}$	0.578* (0.167)	− 0.051** (0.307)	1.213 (1.391)	0.372*** (0.165)	− 0.993 (1.854)	0.681 (0.443)	− 0.152*** (0.139)	− 0.059*** (0.162)
Observations	111,984	136,962	51,795	102,039	73,230	128,098	137,050	136,367
Respondents	24,199	27,310	12,270	22,811	19,598	26,960	27,314	27,278
High education								
$\hat{\beta }_{1}$	0.261*** (0.053)	− 0.014 (0.012)	0.380 (0.584)	0.076*** (0.021)	0.040 (0.060)	0.041 (0.066)	− 0.021 (0.070)	0.035 (0.027)
$\hat{\beta }_{2}$	0.155*** (0.023)	− 0.002 (0.005)	0.332 (0.245)	0.025** (0.009)	0.063* (0.026)	0.020 (0.028)	0.066* (0.030)	− 0.020 (0.012)
$\hat{\beta }_{2} /\hat{\beta }_{1}$	0.592** (0.143)	0.168* (0.404)	0.872 (1.459)	0.335*** (0.149)	1.603 (2.472)	0.490 (1.029)	− 3.163 (10.784)	− 0.575 (0.557)
Observations	47,690	54,973	13,901	44,418	32,153	51,455	54,999	54,744
Respondents	10,040	10,758	3496	9696	8405	10,628	10,759	10,749
Moscow/St Petersburg								
$\hat{\beta }_{1}$	0.439*** (0.107)	0.023 (0.027)	1.352 (1.271)	0.014 (0.045)	0.120 (0.131)	0.284* (0.122)	0.089 (0.147)	0.071 (0.055)
$\hat{\beta }_{2}$	0.226*** (0.037)	0.003 (0.009)	0.407 (0.325)	0.015 (0.015)	0.095* (0.044)	0.122** (0.040)	0.019 (0.048)	− 0.005 (0.018)
$\hat{\beta }_{2} /\hat{\beta }_{1}$	0.515** (0.148)	0.145* (0.410)	0.301 (0.363)	1.103 (3.752)	0.794 (0.915)	0.431* (0.228)	0.216 (0.631)	− 0.065*** (0.256)
Observations	18,054	22,011	8842	16,191	12,716	20,372	22,019	21,896
Respondents	4454	5150	2386	4043	3935	5066	5152	5143
Other regions								
$\hat{\beta }_{1}$	0.213*** (0.039)	− 0.021* (0.009)	0.404 (0.411)	0.086*** (0.016)	− 0.016 (0.047)	0.045 (0.043)	− 0.171** (0.056)	− 0.027 (0.019)
$\hat{\beta }_{2}$	0.129*** (0.017)	− 0.002 (0.004)	0.434* (0.169)	0.026*** (0.007)	0.027 (0.020)	0.031 (0.019)	0.064* (0.025)	− 0.004 (0.009)
$\hat{\beta }_{2} /\hat{\beta }_{1}$	0.606** (0.133)	0.093*** (0.201)	1.073 (1.149)	0.309*** (0.098)	− 1.667 (5.140)	0.704 (0.786)	− 0.376** (0.196)	0.166* (0.344)
Observations	141,620	169,924	56,854	130,266	92,667	159,181	170,030	169,215
Respondents	28,026	30,812	12,859	26,885	22,745	30,473	30,814	30,778

Standard errors in parentheses. The significance tests reported for the $\hat{\beta }_{2} /\hat{\beta }_{1}$ rows tests that the absolute value of the ratio equals one

**p*  < 0.05, ***p*  < 0.01, ****p*  < 0.001

**Table 17:** Number of respondents with self-purchased and workplace provided VHI in Table 15 who answered yes to the question of whether others also paid for their insurance

	Self	Ent
Doctor	5	18
Smoke	6	21
Smoke_n	1	6
Drink	4	14
Drink_n	6	17
Sport	7	21
bmi_dec	7	21
sah	7	21

## Footnotes

1 In fact, there is technically a fourth option as, particularly in the regions, enterprises who cannot enter the corporate VHI sector choose to finance their own medical facilities or send their employees to enterprise owned health resorts or to commission generalised preventative treatment for staff.

2 The RLMS-HSE is a survey conducted by Higher School of Economics and ZAO Demoscope together with Carolina Population Center, University of North Carolina at Chapel Hill and the Institute of Sociology RAS (details at http://www.cpc.unc.edu/projects/rlms(English) and http://www.hse.ru/org/hse/rlms (Russian).

3 Although the official retirement age in Russia remains 55 for women and 60 for men, in the period covered by our data, many Russians continue to work in one or more jobs beyond this official retirement age. We therefore follow the Russian Statistical Agency (ROSSTAT) in identifying 72 as the cut off age for the economically active population.

4 We exclude from our analysis the small number of respondents who report ‘other’ as the source of finance for their VHI and concentrate on the distinction between enterprise funded, self-funded and no VHI.

5 See https://spb.hh.ru/article/24566 and https://tass.ru/obschestvo/6284940 respectively.

6 Taking into account multiple comparisons and the large sample size, there are just two statistically significant results with reliable *p* values: working males who have not been hospitalised in the last year are more likely to have moved into VHI employment, and working females increase their income by moving into VHI employment. This is consistent with no adverse selection.
